# Genetic Diversity of Potassium Ion Channel Proteins Encoded by Chloroviruses That Infect *Chlorella heliozoae*

**DOI:** 10.3390/v12060678

**Published:** 2020-06-23

**Authors:** Carter R. Murry, Irina V. Agarkova, Jayadri S. Ghosh, Fiona C. Fitzgerald, Roger M. Carlson, Brigitte Hertel, Kerri Kukovetz, Oliver Rauh, Gerhard Thiel, James L. Van Etten

**Affiliations:** 1 School of Biological Sciences—Microbiology Program, University of Nebraska-Lincoln, Lincoln, NE 68588-0118, USA; murry.carter12@gmail.com; 2 Nebraska Center for Virology, University of Nebraska-Lincoln, Lincoln, NE 68583-0900, USA; irina@unl.edu (I.V.A.); jghosh2@unl.edu (J.S.G.); roger.carlson@unl.edu (R.M.C.); 3 Department of Plant Pathology, University of Nebraska-Lincoln, Lincoln, NE 68583-0833, USA; 4 Department of Chemistry and Biochemistry, Benedictine College, Atchison, KS 66002, USA; f.fitzgerald559@gmail.com; 5 Membrane Biophysics, Department of Biology, Technische Universitat, 64287 Darmstadt, Germany; hertelb@bio.tu-darmstadt.de (B.H.); kukovetz@bio.tu-darmstadt.de (K.K.); rauh@bio.tu-darmstadt.de (O.R.); thiel@bio.tu-darmstadt.de (G.T.)

**Keywords:** Chloroviruses, potassium ion channels, Kcv channels, algal viruses

## Abstract

Chloroviruses are large, plaque-forming, dsDNA viruses that infect chlorella-like green algae that live in a symbiotic relationship with protists. Chloroviruses have genomes from 290 to 370 kb, and they encode as many as 400 proteins. One interesting feature of chloroviruses is that they encode a potassium ion (K^+^) channel protein named Kcv. The Kcv protein encoded by SAG chlorovirus ATCV-1 is one of the smallest known functional K^+^ channel proteins consisting of 82 amino acids. The Kcv_ATCV-1_ protein has similarities to the family of two transmembrane domain K^+^ channel proteins; it consists of two transmembrane α-helixes with a pore region in the middle, making it an ideal model for studying K^+^ channels. To assess their genetic diversity, *kcv* genes were sequenced from 103 geographically distinct SAG chlorovirus isolates. Of the 103 *kcv* genes, there were 42 unique DNA sequences that translated into 26 new Kcv channels. The new predicted Kcv proteins differed from Kcv_ATCV-1_ by 1 to 55 amino acids. The most conserved region of the Kcv protein was the filter, the turret and the pore helix were fairly well conserved, and the outer and the inner transmembrane domains of the protein were the most variable. Two of the new predicted channels were shown to be functional K^+^ channels.

## 1. Introduction

Chloroviruses (family *Phycodnaviridae*) are large, plaque-forming, dsDNA viruses that infect certain chlorella-like green algae that live in a symbiotic relationship with protists [1]. Chloroviruses have an internal membrane and they are icosahedral in shape with a spike structure at one of their vertices [2]. They have genomes that are 290 to 370 kb in size and are predicted to encode up to 400 proteins (CDSs) and 16 tRNAs. These viruses are ubiquitous in nature and have been isolated from freshwater ponds, lakes, and rivers across the globe. There are four groups of chloroviruses based on the host they infect: viruses that infect *Chlorella variabilis* NC64A (referred to as NC64A viruses), viruses that infect *Chlorella variabilis* Syngen 2-3 (referred to as Osy viruses), viruses that infect *Chlorella heliozoae* SAG 3.83 (referred to as SAG viruses), and viruses that infect *Micratinium conductrix* Pbi, (referred to as Pbi viruses). The most studied chlorovirus is the NC64A virus Paramecium bursaria chlorella virus 1 (PBCV-1); its host, *C. variabilis* NC64A, lives in symbiosis with *Paramecium bursaria*. 

**Figure 1:**
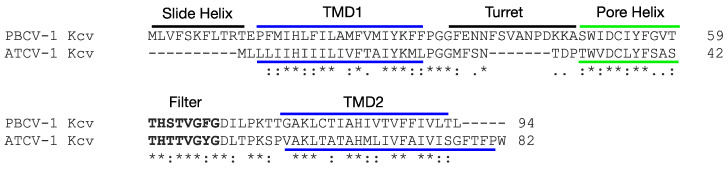
Kcv_ATCV-1_ sequence alignment with the sequence of the prototype chlorovirus K^+^ channel Kcv_PBCV-1_ by ClustalW. The slide helix, outer transmembrane (TMD1) turret, pore helix, selectivity filter, and inner transmembrane (TMD2) of the Kcv_PBCV-1_ are highlighted by the horizontal lines. Asterisks indicate positions which have a fully conserved residue. A colon indicates conservation between amino acids with strongly similar properties. A period indicates conservation between amino acids with less similar properties.

The PBCV-1 genome is ~331 kb and encodes 416 predicted CDSs and 11 tRNA genes. About half of the identified CDSs resemble proteins of known function, including some that are novel for a virus. One protein that PBCV-1, as well as most of the chloroviruses, encodes is a potassium ion (K^+^) channel protein (named Kcv) [3]. When the 94 amino acid Kcv_PBCV-1_ was discovered, it was the smallest protein known to form a functional K^+^ channel. The Kcv_PBCV-1_ protein consists of only the basic functional units that are present in all K^+^ channels in that it has a short slide helix, an outer transmembrane helix, a turret, a pore helix, a filter, and an inner transmembrane helix (Figure 1); four of these proteins form a functional K^+^ channel. 

Kcv_PBCV-1_ is hypothesized to play an important role during infection of its host. After the virus attaches to the host cell wall and degrades the wall at the point of attachment, the PBCV-1 internal membrane fuses with the host’s plasma membrane [4]. The Kcv channel is located in the virus’s internal membrane [5], and once the two membranes are fused, the Kcv channel becomes part of the host membrane. This allows Kcv to participate in the rapid depolarization of the host cell membrane [6,7] and the release of K^+^ from the cell [8].

The rapid loss of K^+^ from the host and associated water fluxes significantly reduce the host turgor pressure, which aids ejection of viral DNA and virion-associated proteins into the host [9]. Host membrane depolarization also inhibits many host secondary transporters [10] and prevents infection by a second virus [11]. Because of the small size of Kcv, it has served as an excellent model for studying K^+^ channels and there are over 60 research publications on Kcv channels.

Since the discovery of Kcv_PBCV-1_, even smaller chlorovirus-encoded K^+^ channel proteins have been described including an 82 amino acid protein from SAG chlorovirus, ATCV-1, referred to as Kcv_ATCV-1_ (Figure 1). Expression studies established that Kcv_ATCV-1_ makes a functional, K^+^ selective channel in *Xenopus laevis* oocytes and in yeast [12]. The objective of this study was to isolate and analyze the sequence diversity of the *kcv* gene from 103 SAG chloroviruses that come from freshwater collected throughout the world. Ultimately, one can anticipate some physiological differences among the Kcv channels from the SAG viruses.

## 2. Materials and Methods 

### 2.1. Cultures

Water samples were collected from lakes, ponds, and rivers from around the United States, Canada, Guatemala, Brazil, Chile, Germany, and Greenland (Table A1 (Appendix A)). The samples were passed through a 0.45 µm filter (PES filters, Sartorius, Gottingen, Germany). Chloroviruses were isolated from the filtered water samples using a plaque assay on a *C. heliozoae* SAG 3.83 lawn. For the plaque assay, plates were made using Modified Bold’s Basal Medium (MBBM) 1.5% agar with tetracycline added at 10 µg/mL [13]. Each plate was filled with about 20 mL of MBBM agar and allowed to solidify. In a tube, 2.5 mL of 0.75% MBBM agar was combined with 1 mL of the filtered water sample and 300 µL of *C. heliozoae* cells (~1.5 × 10^8^ cells/mL). The tube was mixed and poured over the solidified 1.5% MBBM agar plate. Once the top agar layer solidified, the plates were inverted and kept under constant light at 25 °C for a few days. If SAG chloroviruses were present in the water sample, plaques formed on the plates. Two to three unique plaques (e.g., different size and sometimes different shape plaques) were picked from each plate with a sterile toothpick and placed in a 1.5 mL tube filled with *C. heliozoae* cells. These samples were then placed on a spinning wheel for 1 day to propagate the virus. The resulting lysates were serially diluted to 10^−6^ in virus suspension buffer (VSB, 50 mM Tris HCl, 10 mM MgCl_2_, pH 7.8) and 100 µL of the resultant dilution was plaqued. Each virus sample was plaque-purified two or three times to ensure that one had a single virus. For the PCR DNA template preparation, 100 µL of viral lysate was boiled in deionized sterile water for 5–10 min.

### 2.2. Primer Selection

DNA sequences 450 to 500 bp upstream and 230 to 350 bp downstream from the *kcv* gene from 13 previously sequenced SAG chloroviruses [14] were used to identify conserved regions for designing degenerate primers. Conserved regions were identified inside the aligned sequences, and four forward primers and five reverse primers (Table 1) were made and tested using known SAG virus DNAs (ATCV-1, BRO604, Can0610, Canal1, GM0701, MN0810, MO0605, NEJV2, NTS1, OR0704, TN603, WI0606). The primers that identified all of the *kcv* genes were forward primer Kcv8 Frw and reverse primer Kcv6 Rvs as well as forward primer Kcv9 Frw and reverse primer Kcv6 Rvs. As a result, the primer set Kcv9/Kcv6 was selected to amplify the *kcv* genes.

### 2.3. Polymerase Chain Reactions (PCR)

The conditions for the PCR reactions followed the recommendations of New England Biolab’s (Beverly, MA, USA) Phusion High Fidelity DNA Polymerase kit. The 50 µL PCR reactions contained 10 µL of 5x Phusion High Fidelity buffer, 1 U Phusion DNA polymerase, and 1 µL of DNA template. The final concentration of dNTPs in the PCR reaction was 0.2 mM and the primer final concentration was 0.5 µM forward primer and 0.5 µM reverse primer. The reactions were run for 5 min at 94 °C, followed by 35 cycles of 1 min at 94 °C, 30 s at 56 °C, 1 min at 72 °C, and at the end the final extension 15 min at 72 °C. Deionized water was used as a negative control.

The PCR products were gel-purified by mixing 20 µL of an amplified sample with a small amount of Ficoll gel loading buffer and loaded on a 1% agarose gel. The New England Biolabs log-2 ladder was used as a molecular weight marker. The gel was run at 5 V/cm for 1 h, then imaged by exposing the gel to ultraviolet light. Amplified *kcv* genes were excised from the gel and the DNA extracted using the QIAquick Gel Extraction Kit following the manufacturer’s instruction (QIAGEN Hilden, Germany). The purified DNAs were sequenced using Sanger sequencing by a commercial provider. The accession numbers for the *kcv* sequences in GenBank are MT560092-MT560194. 

### 2.4. Phylogenetic Analysis

The *kcv* gene DNA sequences were translated into amino acid sequences, and all of the unique Kcv proteins were aligned. Geneious 11.0.5 software (Biomatters Ltd., Auckland, New Zealand, https://www.geneious.com) was used for the DNA and protein sequence alignment (Geneious Alignment with the default settings) and the phylogenetic tree was constructed with PhyML (version 3.3.20180621), which is Maximum likelihood, using the default settings. 

### 2.5. Functional Reconstitution of kcv Genes in Planar Lipid Bilayers

K^+^ channel proteins were translated in vitro into nanodiscs (NDs) with the MembraneMax HN Protein Expression Kit (Invitrogen, Carlsbad, CA, USA) as described previously [15]. A His-tag attached to the scaffold protein of the NDs allowed purification of channel/ND-complexes via metal chelate affinity chromatography. To eliminate unspecific binders, the column was washed three times with 400 μL of a 20 mM imidazole solution. Finally, the His-tagged NDs were eluted in three fractions with 200 μL of a 250 mM imidazole solution. All centrifugation steps were performed at 700 g for 2 min.

Single-channel recordings were done with a vertical bilayer set up (IonoVation, Osnabrück, Germany) as described previously [16]. The experimental solution contained 100 mM KCl and was buffered to pH 7.0 with 10 mM HEPES/KOH. As a lipid, we used 1,2-diphythanoyl-*sn*-glycero-3-phosphocholine (DPhPC) (Avanti Polar Lipids, Alabaster, AL, USA) at a concentration of 15 mg/mL in n-pentane (MERCK KGaA, Darmstadt, Germany).

## 3. Results

### 3.1. kcv Genes Selected for Analysis

In total, *kcv* genes from 83 SAG chloroviruses were obtained from the PCR experiments. In addition, the *kcv* genes from the 13 SAG chloroviruses that had been sequenced previously [14] and *kcv* genes from 7 recently sequenced SAG chloroviruses (not yet in the database) were included in the analysis. Thus, in total we compared *kcv* genes from 103 SAG chloroviruses (Table A1). Overall, these viruses were from three continents and seven countries.

### 3.2. Diversity of kcv Genes

**Figure A1:**
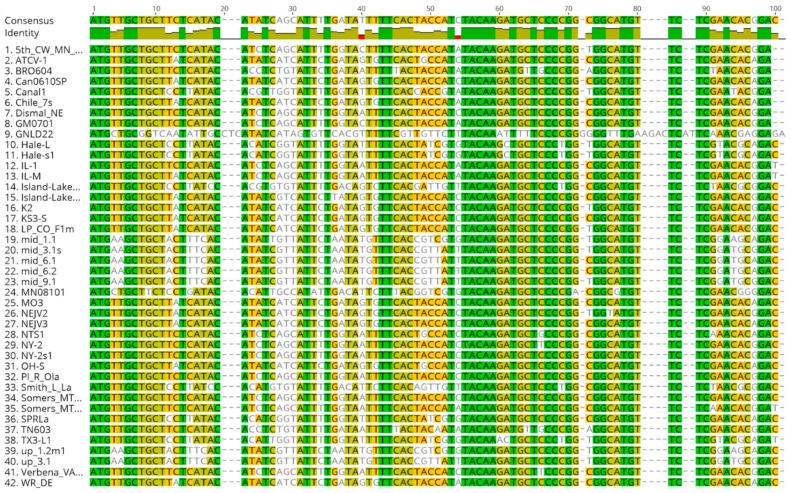

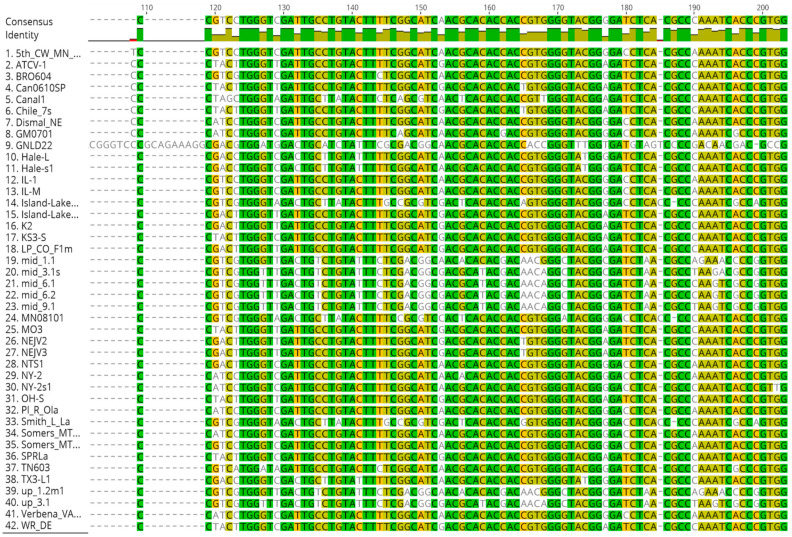

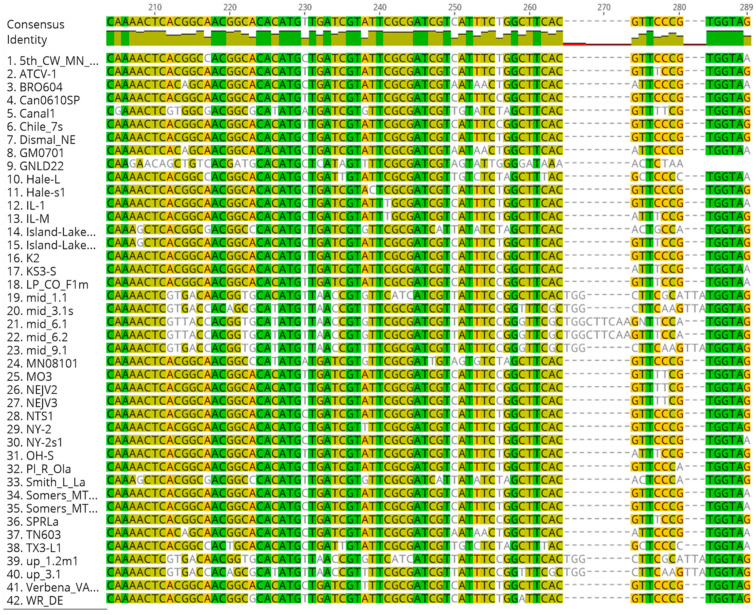
DNA sequence alignment of unique SAG chlorovirus *kcv* genes. DNA sequences were aligned using the Geneious Alignment algorithm from Geneious 11.0.5 software. Green color indicates identical nucletides among all sequenced strains. Different shades from olive to orange denote different degrees of conservation with olive color being the most conserved.

Alignment of the nucleotide sequences indicated that the 103 *kcv* genes had substitutions in 125 of the 249 nucleotides (~50%) (assuming that all the proteins were 82 amino acids long (but see below)) producing 42 unique DNA sequences (Figure A1 (Appendix B)). Nine of the viruses with identical *kcv* DNA sequences included a virus isolated in Germany in 2002 and viruses isolated in various parts of the United States of America 4 to 16 years later. Twenty of the virus isolates with identical *kcv* DNA sequences were from ponds near Hohenheim, Germany that were collected on the same day in 2017. It is important to note that the type SAG virus, ATCV-1, was isolated from one of these ponds in 2002. The sequences of the newly isolated viruses differed from ATCV-1 virus by 18–21 amino acids. In these same recent German collections, there were viruses with three additional *kcv* DNA sequences. The *kcv* DNA sequence from 18 of the 103 viruses was only found one time.

### 3.3. Diversity of the Kcv Proteins

**Figure 2:**
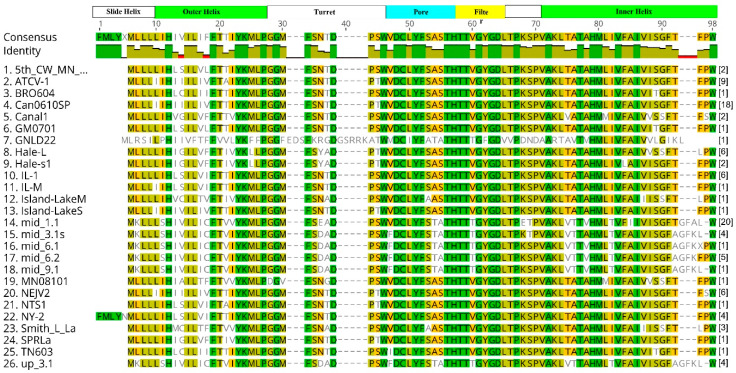
Amino acid alignment of SAG chlorovirus unique Kcv proteins using Geneious 11.0.5 software. The number in brackets at the end of each protein is the number of times that the protein had an identical sequence of the 103 viruses examined. Score matrix is Identity. Green color denotes identical amino acids. Other shades of amino acids indicate a level of conservation from olive color as being the most conserved to white color not conserved.

The 42 unique *kcv* DNA sequences produced 26 unique proteins (Figure 2). Of the 26 unique protein sequences, 18 of them were 82 amino acids long, 4 were 84 amino acids, 2 were 85 amino acids, 1 was 87 amino acids, and 1 was 89 amino acids. The proteins with 84 and 85 amino acids had either 2 or 3 extra amino acids at their C-terminal ends. The 87 amino acid Kcv protein from a virus isolate from New York state had 5 amino acids added to the N-terminus of the protein. However, this protein has an internal Met that would create an 82 amino acid protein and so we suspect that this internal AUG is the actual translation start site. The 89 amino acid protein from a virus, GLND22, isolated in Greenland has 8 extra amino acids in the turret region between the outer transmembrane domain and the pore region. This turret region also has extra amino acids in the slightly larger Kcv proteins from the NC64A viruses (Figure 1) and the Pbi viruses [17].

The Kcv_Can0610SP_ only differed from Kcv_ATCV-1_ by one amino acid. The Kcvs from viruses collected from Germany in 2017 differed from Kcv_ATCV-1,_ which originally came from Germany, by 18 to 21 amino acids. The Kcv_GNLD22_ differed the most from Kcv_ATCV-1_ with 55 amino acid differences or 56% of the amino acids. The remaining virus Kcvs differed from Kcv_ATCV-1_ by 2 to 13 amino acids. 

Alignment of the 26 unique proteins revealed that some areas of the Kcv protein were more conserved than others. The filter domain is typically the most highly conserved domain in K^+^ channel proteins and 20 of the 26 proteins had a TTVGYGDL sequence. Five of the remaining six Kcvs had a TTTGYGDL sequence and the remaining Kcv, Kcv_GLND22_ from Greenland, had a TTTGFGDV sequence (Figure 2). All the SAG chlorovirus Kcvs essentially lack an N-terminal slide helix domain (Figure 1).

**Figure 3:**
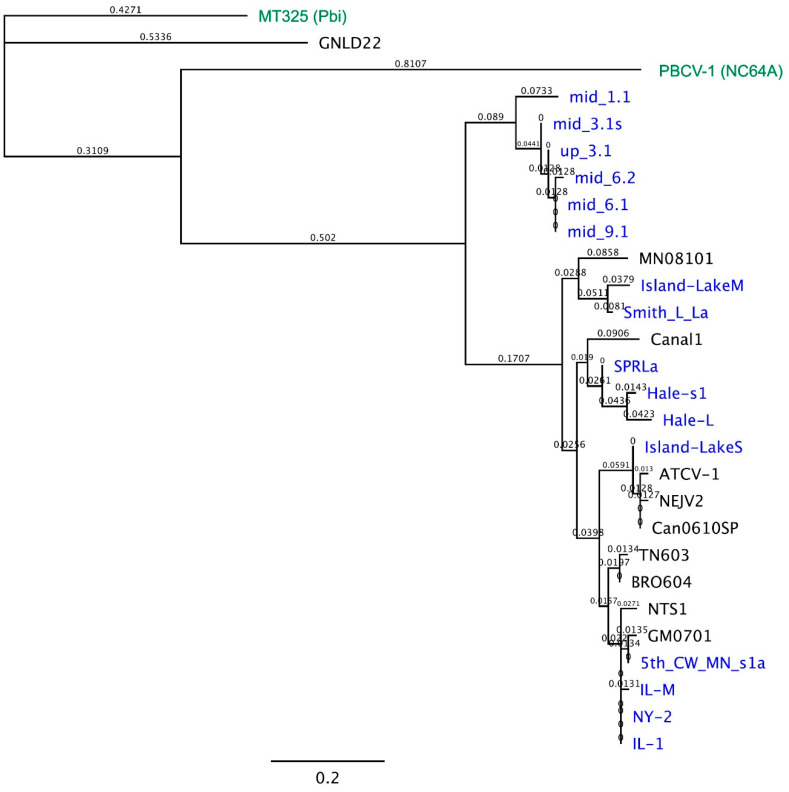
Phylogenetic tree of Kcv proteins from 26 SAG chloroviruses. PhyML, which is Maximum likelihood, with the default settings was used to construct the tree. The branch length shows dissimilarity between strains and the values on the branches are the number of changes. The viruses in blue represent the new Kcv proteins reported in this manuscript. Viruses MT325 representing a Pbi virus and PBCV-1 representing an NC64A virus are in green. Additional information on each of the Kcv proteins from the SAG viruses, including where they were isolated and the number of times that protein sequence appeared, is included in Table A1.

A phylogenetic tree of the 26 Kcv proteins resulted in 3 major clades (Figure 3). The largest clade had 19 Kcv proteins from viruses primarily collected across the United States but also included viruses isolated in Canada, Guatemala, and Brazil, and one from Germany. Kcv proteins isolated from the recent German water samples formed a separate cluster with a distance of 0.4925 substitutions from the rest of the SAG Kcvs (Figure 3). The Kcv_GNDL22_ from Greenland also formed a distinct clade with a distance of 1.518 substitutions from the German samples and a distance of 1.6675 substitutions from all the rest. Interestingly, Kcv_GNDL22_ had more similarity to a Kcv from a Pbi virus MT325 than it did to the other SAG viruses (Figure 3). Virus GNDL22 is also interesting because about 15% of its CDSs are more similar to the MT325 Pbi virus and the other 85% are most similar to SAG viruses. Thus, GNDL22 appears to be some type of hybrid virus. 

### 3.4. Functional Reconstitution of Two New Kcv Channels in Planar Lipid Bilayers

**Figure 4:**
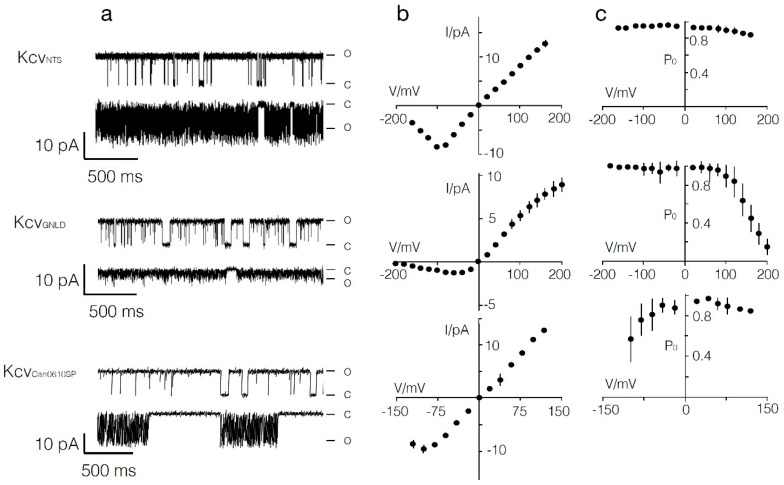
Two of the newly discovered K^+^ channels are active. Characteristic single-channel fluctuations (**a**), mean single-channel I/V relations (**b**), and mean open probabilities (**c**) of Kcv_NTS_ (top row), Kcv_GNLD22_ (middle row), and Kcv_Can0610SP_ (bottom row) at ±120 mV. The closed (c) and open (o) levels are indicated along the current traces. Data are means ±s.d. from ≥ 3 independent recordings of channels in the same row. Data were recorded in a DPhPC bilayer with symmetrical 100 mM KCl, 10 mM HEPES, pH 7 in cis and trans chamber.

To test whether the newly discovered genes were coding for functional K^+^ channels, we selected two representatives for functional testing. The putative channel proteins Kcv_GNLD22_ and Kcv_Can0610SP_ were translated in vitro into nanodiscs and after purification reconstituted in planar lipid bilayers. The electrical recordings in Figure 4 show that the two proteins generated typical single-channel fluctuations at positive and negative voltages in the presence of 100 mM KCl on both sides of the membrane. These experiments established that the two genes code for functional ion channels. The overall properties of the two new channels were similar to those of Kcv_NTS_ (Figure 4), a well-studied representative of the K^+^ channels from SAG viruses [18]; Kcv_NTS_ differed from the reference channel Kcv_ATCV-1_ by four amino acids. Kcv_GNLD22_ and Kcv_Can0610SP_ were selected because of their large (55 amino acids) and small (1 amino acid) deviation from the reference channel Kcv_ATCV-1_. All three channels exhibited a hallmark of the chlorovirus K^+^ channels with well-resolved channel openings at positive voltages and flicker type gating at negative voltages. This flicker type gating resulted from very fast open/close transitions at negative voltages, which cannot be fully resolved by the recording equipment. As a consequence, the unitary channel conductance exhibited an apparent decrease at negative voltages [19,20]. However, it is interesting to note that this fast gating was already apparent in Kcv_GNLD22_ at voltages negative of 0 mV while the negative slope in the two other channels only occurred at voltages more negative than about -100 mV. A closer scrutiny of the single-channel data also showed additional differences between the three channels. Comparison of the unitary channel conductance showed that this value in Kcv_GNLD22_ (45 ± 3 pS, *n* = 7) was only half as big as in Kcv_NTS_ (87 ± 1.4 pS, *n* = 9) and Kcv_Can0610SP_ (110 ± 3, *n* = 9). Another striking feature of Kcv_GNLD22_ was a strong voltage-dependent decrease in open probability at positive voltages, which was not seen in the other two. A peculiar feature of Kcv_Can0610SP_ was long-lived closed states at negative voltages, which explains a voltage-dependent decrease in open probability at negative voltages. These long closures were absent in the two other channels; in Kcv_GNLD22_ it was even difficult to observe any distinct closure at negative voltages. 

## 4. Discussion

This manuscript demonstrated that the *kcv* gene is ubiquitous among the 103 chloroviruses that infect *C. heliozoae* SAG 3.83, which resulted in 42 unique *kcv* DNA sequences. The 42 unique *kcv* DNA sequences produced 26 unique proteins or 26 new Kcv channels. Using the Kcv_ATCV-1_ channel as representative of the SAG viruses, the Kcv_ATCV-1_ differed from the Kcv_GNLD22_ channel from Greenland by 55 amino acids or 56% of their amino acids. Other channels differ from the Kcv_ATCV-1_ channel by 1 to 21 amino acids. 

Due to the role K^+^ channels play in chlorovirus infection and reproduction by depolarizing the host cell membrane, it is not surprising that *kcv* genes were found in all of the samples [19]. Therefore, we predict that all of the amino acid substitutions in the channels from the SAG viruses will produce functional channels; in fact, the two channels that were tested were functional (Figure 4). Furthermore, we predict that the SAG Kcv channels may have some different biophysical properties, especially the Kcv coded by the GNDL22 virus. This prediction is confirmed by scrutiny of the functional properties of the two new channels and a comparison with the well-studied SAG-type channel Kcv_NTS_ [18]. The mutual comparisons identified differences in the unitary conductance and gating between the different channels. The apparent impact of a few amino acid exchanges between the proteins on functional properties is in good agreement with previous investigations in which we found that mutation of the common Gly at the end of the second transmembrane domain in Kcv_NTS_ to Ser introduces one distinct gate with a long-lived close time [18]. Several of the new channels have the same critical Ser in the same position (90 in Figure 2). Thus, a functional analysis of these channels will be interesting. 

The data are also in line with a previous study where the *kcv* gene was sequenced from 41 NC64A viruses, including the prototype chlorovirus PBCV-1. Sixteen of the 94 amino acids in the NC64A Kcv protein differed resulting in six new Kcv channels [20]. The six Kcv-like channels, which differed from Kcv_PBCV-1_ by 4 to 12 amino acids, produced K^+^ selective currents in *Xenopus laevis* oocytes with altered biophysical properties, including current kinetics, voltage dependency, and inhibition by Cs^+^ [20,21]. The amino acid changes together with the different properties observed in the six Kcv-like channels were used to guide site-directed mutations, either singularly or in combination, to identify key amino acids that confer specific properties to Kcv [20,21]. 

While we assume that the chloroviruses require Kcv activity to replicate, we do have to mention that out of the more than 150 chloroviruses that have been examined for a *kcv* gene, two NC64A viruses either lack a *kcv* gene or have a truncated form [1]. We would like to disrupt the *kcv* gene in some of the chloroviruses to see what effect this has on virus replication, however, currently the technology to do this experiment is not available. 

Compared with the larger Kcv_PBCV-1_, the 82 amino acid Kcv_ATCV-1_ lacks a cytoplasmic N-terminus, that is, the slide helix region and a number of charged amino acids in its turret domain (Figure 1) [12]. The only known K^+^ channel proteins smaller than Kcv_ATCV-1_ are two channels that are encoded by viruses that infect small marine algae in the *Micromonas* genus [22]. The channel Kmbv_1_ is 79 amino acids long and Kmpv_12T_ is 78 amino acids long. Expression of Kmbv_1_ in HEK293 cells results in currents. However, expression of Kmpv_12T_ in the same cells does not produce a current, but it does produce a current in a planar lipid bilayer [22]. All of these virus-encoded channels have a long evolutionary history and probably have a common evolutionary ancestor.

In summary, *kcv* genes from 103 geographically distinct SAG viruses were sequenced to assess their genetic diversity. Of the 103 *kcv* genes, there were 42 unique DNA sequences that translated into 26 new Kcv proteins, which we predict will have some different biophysical properties. The amino acid changes together with the expected different properties will be used to guide site-directed mutations to identify key amino acids that confer specific properties to Kcv.

## Figures and Tables

**Table 1:** Primers tested to isolate the *kcv* genes.

	Name	Sequence	Position	GC Content (%)	T_m_ (°C)
Forward Primers	Kcv6 Frw	CTT TAG YYT TYY TCK GVC	−366	34	49
	Kcv7 Frw	CTT TAG YYT TYY TCK GVC G	−366	38	54
	Kcv8 Frw	GAA GCA GGY ACC ACT TTA G	−379	47	53
	Kcv9 Frw	GCA GGY ACC ACT TTA G	−376	50	47
Reverse Primers	Kcv6 Rvs	CRC RGM ATR TRT CAT TTG WCC C	+256	48	64
	Kcv7 Rvs	CTT ACR CRG MAT RTR TCA TTT G	+259	39	56
	Kcv8 Rvs	CTT ACR CRG MAT RTR TC	+264	44	44
	Kcv9 Rvs	HKB YMC GAT CTT ATA CAC	+292	39	45
	Kcv10 Rvs	CAT TTC TTA CRC RGM ATR TRT C	+264	39	55

## Appendix A

**Table A1:** Source and sequence of SAG chlorovirus encoded potassium ion channel (Kcv) genes ^1^.

Sample ID	DNA Sequence	Location collected	Date collected	DNA Group	Amino Acid Group
5th_CW_MN_s1a (2) [2]	ATGTTGCTGCTTCTCATACATCTCAGCATTTTGGTACTTTTCACTACCATATACAAGATGCTCCCCGGTGGCATGTTCTCGAACACGGATCCGTCCTGGGTCGATTGCCTGTACTTTTCGGCATCAACGCACACCACCGTGGGGTACGGGGACCTCACGCCAAAATCACCCGTGGCAAAACTCACGGCCACGGCACACATGCTGATCGTATTCGCGATCGTCATTTCTGGCTTCACGTTCCCGTGGTAA	5th Crow Wing Lake; Nevis, Minnesota	5/4/2017	5th_CW_MN_s1a	5th_CW_MN_s1a
5th_CW_MN_L4	ATGTTGCTGCTTCTCATACATCTCAGCATTTTGGTACTTTTCACTACCATATACAAGATGCTCCCCGGTGGCATGTTCTCGAACACGGATCCGTCCTGGGTCGATTGCCTGTACTTTTCGGCATCAACGCACACCACCGTGGGGTACGGGGACCTCACGCCAAAATCACCCGTGGCAAAACTCACGGCCACGGCACACATGCTGATCGTATTCGCGATCGTCATTTCTGGCTTCACGTTCCCGTGGTAA	5th Crow Wing Lake; Nevis, Minnesota	5/4/2017	5th_CW_MN_s1a	5th_CW_MN_s1a
ATCV-1 (8) [9]	ATGTTGCTGCTTATCATACATATCATCATTCTGATAGTGTTCACTGCCATCTACAAGATGCTCCCCGGCGGCATGTTCTCGAACACAGACCCTACTTGGGTTGATTGCCTGTACTTTTCGGCATCGACGCACACCACCGTGGGGTACGGAGATCTCACGCCCAAATCACCCGTGGCAAAACTCACGGCAACGGCACACATGTTGATCGTATTCGCGATCGTCATTTCTGGCTTCACGTTTCCGTGGTAG	Germany	2002	ATCV-1	ATCV-1
MO0605SPH	ATGTTGCTGCTTATCATACATATCATCATTCTGATAGTGTTCACTGCCATCTACAAGATGCTCCCCGGCGGCATGTTCTCGAACACAGACCCTACTTGGGTTGATTGCCTGTACTTTTCGGCATCGACGCACACCACCGTGGGGTACGGAGATCTCACGCCCAAATCACCCGTGGCAAAACTCACGGCAACGGCACACATGTTGATCGTATTCGCGATCGTCATTTCTGGCTTCACGTTTCCGTGGTAG	Missouri	2006	ATCV-1	ATCV-1
WI0606	ATGTTGCTGCTTATCATACATATCATCATTCTGATAGTGTTCACTGCCATCTACAAGATGCTCCCCGGCGGCATGTTCTCGAACACAGACCCTACTTGGGTTGATTGCCTGTACTTTTCGGCATCGACGCACACCACCGTGGGGTACGGAGATCTCACGCCCAAATCACCCGTGGCAAAACTCACGGCAACGGCACACATGTTGATCGTATTCGCGATCGTCATTTCTGGCTTCACGTTTCCGTGGTAG	Madison, Wisconsin	2006	ATCV-1	ATCV-1
Drexel	ATGTTGCTGCTTATCATACATATCATCATTCTGATAGTGTTCACTGCCATCTACAAGATGCTCCCCGGCGGCATGTTCTCGAACACAGACCCTACTTGGGTTGATTGCCTGTACTTTTCGGCATCGACGCACACCACCGTGGGGTACGGAGATCTCACGCCCAAATCACCCGTGGCAAAACTCACGGCAACGGCACACATGTTGATCGTATTCGCGATCGTCATTTCTGGCTTCACGTTTCCGTGGTAG	Drexel, Missouri	Jun-17	ATCV-1	ATCV-1
NPRLb	ATGTTGCTGCTTATCATACATATCATCATTCTGATAGTGTTCACTGCCATCTACAAGATGCTCCCCGGCGGCATGTTCTCGAACACAGACCCTACTTGGGTTGATTGCCTGTACTTTTCGGCATCGACGCACACCACCGTGGGGTACGGAGATCTCACGCCCAAATCACCCGTGGCAAAACTCACGGCAACGGCACACATGTTGATCGTATTCGCGATCGTCATTTCTGGCTTCACGTTTCCGTGGTAG	North Platte River; Highway 27, Nebraska	10/23/2017	ATCV-1	ATCV-1
SPRma	ATGTTGCTGCTTATCATACATATCATCATTCTGATAGTGTTCACTGCCATCTACAAGATGCTCCCCGGCGGCATGTTCTCGAACACAGACCCTACTTGGGTTGATTGCCTGTACTTTTCGGCATCGACGCACACCACCGTGGGGTACGGAGATCTCACGCCCAAATCACCCGTGGCAAAACTCACGGCAACGGCACACATGTTGATCGTATTCGCGATCGTCATTTCTGGCTTCACGTTTCCGTGGTAG	South Platte River; Big Spring, Nebraska	10/23/2017	ATCV-1	ATCV-1
SPRsb	ATGTTGCTGCTTATCATACATATCATCATTCTGATAGTGTTCACTGCCATCTACAAGATGCTCCCCGGCGGCATGTTCTCGAACACAGACCCTACTTGGGTTGATTGCCTGTACTTTTCGGCATCGACGCACACCACCGTGGGGTACGGAGATCTCACGCCCAAATCACCCGTGGCAAAACTCACGGCAACGGCACACATGTTGATCGTATTCGCGATCGTCATTTCTGGCTTCACGTTTCCGTGGTAG	South Platte River; Big Spring, Nebraska	10/23/2017	ATCV-1	ATCV-1
Smith-Lake_Large-Plaque	ATGTTGCTGCTTATCATACATATCATCATTCTGATAGTGTTCACTGCCATCTACAAGATGCTCCCCGGCGGCATGTTCTCGAACACAGACCCTACTTGGGTTGATTGCCTGTACTTTTCGGCATCGACGCACACCACCGTGGGGTACGGAGATCTCACGCCCAAATCACCCGTGGCAAAACTCACGGCAACGGCACACATGTTGATCGTATTCGCGATCGTCATTTCTGGCTTCACGTTTCCGTGGTAG	Smith Lake, Western Nebraska	2018	ATCV-1	ATCV-1
OH-S (1)	ATGTTGCTGCTTATCATACATATCATCATTCTGATAGTGTTCACTGCCATCTACAAGATGCTCCCCGGCGGCATGTTCTCGAACACAGACCCTACTTGGGTTGATTGCCTGTACTTTTCGGCATCGACGCACACCACCGTGGGGTACGGAGATCTCACGCCCAAATCACCCGTGGCAAAACTCACGGCAACGGCACACATGTTGATCGTATTCGCGATCGTCATTTCTGGCTTCACATTTCCGTGGTAG	Ohio	Jul-17	OH-S	ATCV-1
BRO604 (1) [1]	ATGTTGCTGCTTCTCATACACCTCTGTATTCTGATAATTTTTACTACCATATACAAGATGTTGCCCGGAGGCATGTTCTCTAACACGGACCCGTCGTGGGTCGATTGCCTGTACTTCTCGGCATCAACGCACACCACCGTGGGGTACGGGGATCTCACGCCCAAATCACCCGTGGCAAAACTCACAGCAACGGCACACATGCTGATCGTATTCGCGATCGTAATAACTGGCTTCACATTCCCGTGGTAA	Brazil	2006	BRO604	BRO604
Can0610SP (2) [18]	ATGTTGCTGCTTATCATACATATCATCATTCTGATAGTGTTCACTACCATCTACAAGATGCTCCCCGGCGGCATGTTCTCGAACACAGACCCTACTTGGGTTGATTGCCTGTACTTTTCGGCATCGACGCACACCACTGTGGGGTACGGAGATCTCACGCCCAAATCACCCGTGGCAAAACTCACGGCAACGGCACACATGTTGATCGTATTCGCGATCGTCATTTCCGGCTTCACGTTCCCGTGGTAG	British Columbia, Canada	2006	Can0610SP	Can0610SP
Or0704	ATGTTGCTGCTTATCATACATATCATCATTCTGATAGTGTTCACTACCATCTACAAGATGCTCCCCGGCGGCATGTTCTCGAACACAGACCCTACTTGGGTTGATTGCCTGTACTTTTCGGCATCGACGCACACCACTGTGGGGTACGGAGATCTCACGCCCAAATCACCCGTGGCAAAACTCACGGCAACGGCACACATGTTGATCGTATTCGCGATCGTCATTTCCGGCTTCACGTTCCCGTGGTAG	Willamette River; Corvallis, Oregon	Jul-07	Can0610SP	Can0610SP
Chile_7s (1)	ATGTTGCTGCTTATCATACATATCATCATTCTGATAGTGTTCACTACCATATACAAGATGCTCCCCGGCGGCATGTTCTCGAACACAGACCCTACTTGGGTTGATTGCCTGTACTTTTCGGCATCGACGCACACCACTGTGGGGTACGGAGATCTCACGCCCAAATCACCCGTGGCAAAACTCACGGCAACGGCACACATGTTGATCGTATTCGCGATCGTCATTTCCGGCTTCACGTTCCCGTGGTAG	Chile	Jan-19	Chile_7s	Can0610SP
K2 (5)	ATGTTGCTGCTTATCATACATATCATCATTCTGATAGTGTTCACTACCATCTACAAGATGCTCCCCGGTGGCATGTTCTCGAACACGGACCCGACTTGGGTTGATTGCCTGTACTTTTCGGCATCGACGCACACCACCGTGGGGTACGGAGATCTCACGCCCAAATCACCCGTGGCAAAACTCACGGCAACGGCACACATGTTGATCGTATTCGCGATCGTCATTTCCGGCTTCACGTTCCCGTGGTAG	Missouri River; Atchison, Kansas	5/31/2017	K2	Can0610SP
K2s	ATGTTGCTGCTTATCATACATATCATCATTCTGATAGTGTTCACTACCATCTACAAGATGCTCCCCGGTGGCATGTTCTCGAACACGGACCCGACTTGGGTTGATTGCCTGTACTTTTCGGCATCGACGCACACCACCGTGGGGTACGGAGATCTCACGCCCAAATCACCCGTGGCAAAACTCACGGCAACGGCACACATGTTGATCGTATTCGCGATCGTCATTTCCGGCTTCACGTTCCCGTGGTAG	Missouri River; Atchison, Kansas	5/31/2017	K2	Can0610SP
NPRma	ATGTTGCTGCTTATCATACATATCATCATTCTGATAGTGTTCACTACCATCTACAAGATGCTCCCCGGTGGCATGTTCTCGAACACGGACCCGACTTGGGTTGATTGCCTGTACTTTTCGGCATCGACGCACACCACCGTGGGGTACGGAGATCTCACGCCCAAATCACCCGTGGCAAAACTCACGGCAACGGCACACATGTTGATCGTATTCGCGATCGTCATTTCCGGCTTCACGTTCCCGTGGTAG	North Platte River; Highway 27, Nebraska	10/23/2017	K2	Can0610SP
NPRsb	ATGTTGCTGCTTATCATACATATCATCATTCTGATAGTGTTCACTACCATCTACAAGATGCTCCCCGGTGGCATGTTCTCGAACACGGACCCGACTTGGGTTGATTGCCTGTACTTTTCGGCATCGACGCACACCACCGTGGGGTACGGAGATCTCACGCCCAAATCACCCGTGGCAAAACTCACGGCAACGGCACACATGTTGATCGTATTCGCGATCGTCATTTCCGGCTTCACGTTCCCGTGGTAG	North Platte River; Highway 27, Nebraska	10/23/2017	K2	Can0610SP
Pl_R_Lma	ATGTTGCTGCTTATCATACATATCATCATTCTGATAGTGTTCACTACCATCTACAAGATGCTCCCCGGTGGCATGTTCTCGAACACGGACCCGACTTGGGTTGATTGCCTGTACTTTTCGGCATCGACGCACACCACCGTGGGGTACGGAGATCTCACGCCCAAATCACCCGTGGCAAAACTCACGGCAACGGCACACATGTTGATCGTATTCGCGATCGTCATTTCCGGCTTCACGTTCCCGTGGTAG	Platte River; Louisville, Nebraska	10/26/2017	K2	Can0610SP
KS3-S (1)	ATGTTGCTGCTTATCATACATATCATCATTCTGATAGTGTTCACTACCATCTACAAGATGCTCCCCGGCGGCATGTTCTCGAACACAGACCCTACTTGGGTCGATTGCCTGTACTTTTCGGCATCGACGCACACCACCGTGGGGTACGGAGATCTCACGCCCAAATCACCCGTGGCAAAACTCACGGCAACGGCACACATGTTGATCGTATTCGCGATCGTCATTTCCGGCTTCACATTTCCGTGGTAA	Delaware River; Valley Fall, Kansas	Jun-17	KS3-S	Can0610SP
LP_CO_F1m (7)	ATGTTGCTGCTTATCATACATATCATCATTCTGATAGTGTTCACTACCATCTACAAGATGCTCCCCGGTGGCATGTTCTCGAACACGGACCCGACTTGGGTTGATTGCCTGTACTTTTCGGCATCGACGCACACCACCGTGGGGTACGGAGATCTCACGCCCAAATCACCCGTGGCAAAACTCACGGCAACGGCACACATGTTGATCGTATTCGCGATCGTCATTTCCGGCTTCACGTTTCCGTGGTAA	Cache la Poudre River, Colorado	5/29/2017	LP_CO_F1m	Can0610SP
LP_CO_F2L	ATGTTGCTGCTTATCATACATATCATCATTCTGATAGTGTTCACTACCATCTACAAGATGCTCCCCGGTGGCATGTTCTCGAACACGGACCCGACTTGGGTTGATTGCCTGTACTTTTCGGCATCGACGCACACCACCGTGGGGTACGGAGATCTCACGCCCAAATCACCCGTGGCAAAACTCACGGCAACGGCACACATGTTGATCGTATTCGCGATCGTCATTTCCGGCTTCACGTTTCCGTGGTAA	Cache la Poudre River, Colorado	5/30/2017	LP_CO_F1m	Can0610SP
LP_CO_F2m	ATGTTGCTGCTTATCATACATATCATCATTCTGATAGTGTTCACTACCATCTACAAGATGCTCCCCGGTGGCATGTTCTCGAACACGGACCCGACTTGGGTTGATTGCCTGTACTTTTCGGCATCGACGCACACCACCGTGGGGTACGGAGATCTCACGCCCAAATCACCCGTGGCAAAACTCACGGCAACGGCACACATGTTGATCGTATTCGCGATCGTCATTTCCGGCTTCACGTTTCCGTGGTAA	Cache la Poudre River, Colorado	5/31/2017	LP_CO_F1m	Can0610SP
LP_CO_F2s	ATGTTGCTGCTTATCATACATATCATCATTCTGATAGTGTTCACTACCATCTACAAGATGCTCCCCGGTGGCATGTTCTCGAACACGGACCCGACTTGGGTTGATTGCCTGTACTTTTCGGCATCGACGCACACCACCGTGGGGTACGGAGATCTCACGCCCAAATCACCCGTGGCAAAACTCACGGCAACGGCACACATGTTGATCGTATTCGCGATCGTCATTTCCGGCTTCACGTTTCCGTGGTAA	Cache la Poudre River, Colorado	6/1/2017	LP_CO_F1m	Can0610SP
LP_CO_F3L	ATGTTGCTGCTTATCATACATATCATCATTCTGATAGTGTTCACTACCATCTACAAGATGCTCCCCGGTGGCATGTTCTCGAACACGGACCCGACTTGGGTTGATTGCCTGTACTTTTCGGCATCGACGCACACCACCGTGGGGTACGGAGATCTCACGCCCAAATCACCCGTGGCAAAACTCACGGCAACGGCACACATGTTGATCGTATTCGCGATCGTCATTTCCGGCTTCACGTTTCCGTGGTAA	Cache la Poudre River, Colorado	6/2/2017	LP_CO_F1m	Can0610SP
LP_CO_F3m	ATGTTGCTGCTTATCATACATATCATCATTCTGATAGTGTTCACTACCATCTACAAGATGCTCCCCGGTGGCATGTTCTCGAACACGGACCCGACTTGGGTTGATTGCCTGTACTTTTCGGCATCGACGCACACCACCGTGGGGTACGGAGATCTCACGCCCAAATCACCCGTGGCAAAACTCACGGCAACGGCACACATGTTGATCGTATTCGCGATCGTCATTTCCGGCTTCACGTTTCCGTGGTAA	Cache la Poudre River, Colorado	6/3/2017	LP_CO_F1m	Can0610SP
LP_CO_F4s	ATGTTGCTGCTTATCATACATATCATCATTCTGATAGTGTTCACTACCATCTACAAGATGCTCCCCGGTGGCATGTTCTCGAACACGGACCCGACTTGGGTTGATTGCCTGTACTTTTCGGCATCGACGCACACCACCGTGGGGTACGGAGATCTCACGCCCAAATCACCCGTGGCAAAACTCACGGCAACGGCACACATGTTGATCGTATTCGCGATCGTCATTTCCGGCTTCACGTTTCCGTGGTAA	Cache la Poudre River, Colorado	6/4/2017	LP_CO_F1m	Can0610SP
WR_DE (2)	ATGTTGCTGCTTATCATACATATCATCATTCTGATAGTGTTCACTACCATATACAAGATGCTCCCCGGCGGCATGTTCTCGAACACAGACCCTACTTGGGTCGATTGCCTGTACTTTTCGGCATCGACGCACACCACCGTGGGGTACGGAGATCTCACGCCCAAATCACCCGTGGCAAAACTCACGGCAACGGCGCACATGTTGATCGTATTCGCGATCGTCATTTCTGGATTCACGTTCCCGTGGTAG	Wilson Run: Winterhur, Delaware	Jul-17	WR_DE	Can0610SP
WR_DE_s2cr2	ATGTTGCTGCTTATCATACATATCATCATTCTGATAGTGTTCACTACCATATACAAGATGCTCCCCGGCGGCATGTTCTCGAACACAGACCCTACTTGGGTCGATTGCCTGTACTTTTCGGCATCGACGCACACCACCGTGGGGTACGGAGATCTCACGCCCAAATCACCCGTGGCAAAACTCACGGCAACGGCGCACATGTTGATCGTATTCGCGATCGTCATTTCTGGATTCACGTTCCCGTGGTAG	Wilson Run, Winterthur; Delaware	Jul-17	WR_DE	Can0610SP
Canal1 (2) [2]	ATGTTGCTGCTCCTTATACACGTTGGTATTTTGGTATTTTTCACCACCGTATACAAGATGCTCCCCGGTGGCATGTTCTCGAATACGGACCCTAGCTGGGTAGATTGCTTATACTTCTCAGCGTCAACTCACACCACCGTTGGGTACGGAGATCTCACGCCCAAATCACCCGTGGCGAAACTCGTGGCGACGGCGCATATGATGATCGTGTTCGCGATCGTTGTATCTAGCTTCACGTTTTCGTGGTAG	Canal exiting Smith Lake, Nebraska	2008	Canal1	Canal1
Smith-Lake_Small-Plaque	ATGTTGCTGCTCCTTATACACGTTGGTATTTTGGTATTTTTCACCACCGTATACAAGATGCTCCCCGGTGGCATGTTCTCGAATACGGACCCTAGCTGGGTAGATTGCTTATACTTCTCAGCGTCAACTCACACCACCGTTGGGTACGGAGATCTCACGCCCAAATCACCCGTGGCGAAACTCGTGGCGACGGCGCATATGATGATCGTGTTCGCGATCGTTGTATCTAGCTTCACGTTTTCGTGGTAG	Smith Lake, Western Nebraska	2018	Canal1	Canal1
GM0701 (1) [1]	ATGTTGCTGCTTCTCATACATCTCAGCATTTTGGTACTTTTCACTACCATATACAAGATGCTCCCCGGCGGCATGTTCTCGAACACGGACCCATCCTGGGTCGATTGCCTGTACTTTTCAGCATCAACGCACACGACCGTGGGGTACGGGGACCTCACGCCAAAATCGCCCGTGGCAAAACTCACAGCAACGGCACACATGCTGATCGTATTCGCGATCGTAATAACTGGCTTCACATTCCCGTGGTAA	Guatemala	2007	GM0701	GM0701
GNLD22 (1) [1]	ATGCTGCGGTCAATATTGCCTCATATCATAGTGTTCACGTTTTTCGTTGTTCTTTACAAATTTTTCCCCGGGGGGTTTGAAGACTCATTCAAACGAGGAGACGGGTCCCGCAGAAAGGCGACGTGGATGGACTGCATCTATTTCGCGACGGCAACGCACACCACCACCGGGTTTGGTGATGTAGTCCCCGACAACGACGCCGCAAGAACAGCTGTCACGATGCACATGCTCATAGTTTTCGCGATCGTAGTATTGGGGATAAAACTCTAA	Lake Sisimiut, Greenland	2012	GNLD22	GNLD22
Hale-L (2) [6]	ATGTTGCTGCTCCTTATACACATCGGTATTTTGGTATTTTTCACTATCGTGTACAAGCTGCTCCCTGGTGGCATGTTCTCGTACGCAGACCCGACCTGGGTCGACTGCTTGTATTTTTCGGCATCAACGCACACCACCGTGGGGTATGGGGATCTCACGCCCAAATCACCCGTGGCAAAACTCACGGCCACGGCACACATGCTGATTGTATTCGCGATCGTTGTCTCTAGCTTTACGCTCCCCTGGTAA	North branch of Elk Creek; Hale, Wisconsin	7/4/2017	Hale-L	Hale-L
Hale_WI_m4	ATGTTGCTGCTCCTTATACACATCGGTATTTTGGTATTTTTCACTATCGTGTACAAGCTGCTCCCTGGTGGCATGTTCTCGTACGCAGACCCGACCTGGGTCGACTGCTTGTATTTTTCGGCATCAACGCACACCACCGTGGGGTATGGGGATCTCACGCCCAAATCACCCGTGGCAAAACTCACGGCCACGGCACACATGCTGATTGTATTCGCGATCGTTGTCTCTAGCTTTACGCTCCCCTGGTAA	North branch of Elk Creek; Hale, Wisconsin	7/4/2017	Hale-L	Hale-L
TX3-L1 (4)	ATGTTGCTGCTCCTTATACACATTGGTATTTTGGTATTTTTCACTATCGTGTACAAACTGCTCCCTGGTGGCATGTTCTCGTACGCAGATCCGACCTGGGTCGACTGCTTGTATTTTTCGGCATCAACGCACACCACCGTGGGGTATGGGGATCTCACGCCCAAATCACCCGTGGCAAAACTCACGGCCACTGCACACATGCTGATTGTATTCGCGATCGTTGTCTCTAGCTTTACGCTCCCCTGGTAA	Colorado River Pond; Austin, Texas	Jul-17	TX3-L1	Hale-L
TX3-L2	ATGTTGCTGCTCCTTATACACATTGGTATTTTGGTATTTTTCACTATCGTGTACAAACTGCTCCCTGGTGGCATGTTCTCGTACGCAGATCCGACCTGGGTCGACTGCTTGTATTTTTCGGCATCAACGCACACCACCGTGGGGTATGGGGATCTCACGCCCAAATCACCCGTGGCAAAACTCACGGCCACTGCACACATGCTGATTGTATTCGCGATCGTTGTCTCTAGCTTTACGCTCCCCTGGTAA	Colorado River Pond, Austin, Texas	Jul-17	TX3-L1	Hale-L
TX3m	ATGTTGCTGCTCCTTATACACATTGGTATTTTGGTATTTTTCACTATCGTGTACAAACTGCTCCCTGGTGGCATGTTCTCGTACGCAGATCCGACCTGGGTCGACTGCTTGTATTTTTCGGCATCAACGCACACCACCGTGGGGTATGGGGATCTCACGCCCAAATCACCCGTGGCAAAACTCACGGCCACTGCACACATGCTGATTGTATTCGCGATCGTTGTCTCTAGCTTTACGCTCCCCTGGTAA	Colorado River Pond; Austin, Texas	Jul-17	TX3-L1	Hale-L
TX3s	ATGTTGCTGCTCCTTATACACATTGGTATTTTGGTATTTTTCACTATCGTGTACAAACTGCTCCCTGGTGGCATGTTCTCGTACGCAGATCCGACCTGGGTCGACTGCTTGTATTTTTCGGCATCAACGCACACCACCGTGGGGTATGGGGATCTCACGCCCAAATCACCCGTGGCAAAACTCACGGCCACTGCACACATGCTGATTGTATTCGCGATCGTTGTCTCTAGCTTTACGCTCCCCTGGTAA	Colorado River Pond; Austin, Texas	Jul-17	TX3-L1	Hale-L
Hale-s1 (2) [2]	ATGTTGCTGCTCCTTATACACATCGGTATTTTGGTATTTTTCACTATCGTGTACAAGCTGCTCCCTGGTGGCATGTTCTCGTACGCAGACCCGACCTGGGTCGACTGCTTGTATTTTTCGGCATCAACGCACACCACCGTGGGGTATGGGGATCTCACGCCCAAATCACCCGTGGCAAAACTCACGGCAACGGCACACATGCTGATCGTACTCGCGATCGTCATTTCTGGCTTCACGTTCCCGTGGTAG	North branch of Elk Creek; Hale, Wisconsin	7/4/2017	Hale-s1	Hale-s1
Hale-s2	ATGTTGCTGCTCCTTATACACATCGGTATTTTGGTATTTTTCACTATCGTGTACAAGCTGCTCCCTGGTGGCATGTTCTCGTACGCAGACCCGACCTGGGTCGACTGCTTGTATTTTTCGGCATCAACGCACACCACCGTGGGGTATGGGGATCTCACGCCCAAATCACCCGTGGCAAAACTCACGGCAACGGCACACATGCTGATCGTACTCGCGATCGTCATTTCTGGCTTCACGTTCCCGTGGTAG	North branch of Elk Creek; Hale, Wisconsin	7/4/2017	Hale-s1	Hale-s1
IL-1 (1) [6]	ATGTTGCTGCTTCTCATACATCTCAGCATTTTGGTAATTTTCACTACCATATACAAGATGCTCCCCGGCGGCATGTTCTCGAACACGGATCCGTCCTGGGTCGATTGCCTGTACTTTTCGGCATCAACGCACACCACCGTGGGGTACGGGGACCTCACGCCAAAATCACCCGTGGCAAAACTCACGGCAACGGCACACATGCTGATCGTATTTGCGATCGTCATTTCTGGCTTCACGTTCCCGTGGTAA	Lake Zurich, Illinois	6/27/2017	IL-1	IL-1
Dismal_NE (1)	ATGTTGCTGCTTCTCATACATCTCAGCATTTTGGTAATTTTCACTACCATCTACAAGATGCTCCCCGGCGGCATGTTCTCGAACACGGACCCATCCTGGGTCGATTGCCTGTACTTTTCGGCATCAACGCACACCACCGTGGGGTACGGGGACCTCACGCCAAAATCACCCGTGGCAAAACTCACGGCAACGGCACACATGCTGATCGTATTCGCGATCGTCATTTCTGGCTTCACGTTCCCGTGGTAG	Dismal River, Nebraska	5/26/2017	Dismal_NE	IL-1
NY2s1 (2)	ATGTTGCTGCTTCTCATACATCTCAGCATTTTGGTAATTTTCACTACCATCTACAAGATGCTCCCCGGCGGCATGTTCTCGAACACGGACCCATCCTGGGTCGATTGCCTGTACTTTTCGGCATCAACGCACACCACCGTGGGGTACGGGGACCTCACGCCCAAATCACCCGTTGCAAAACTCACGGCAACGGCACACATGCTGATCGTATTCGCGATCGTCATTTCTGGCTTCACGTTCCCGTGGTAA	Moodna Creek; Washingtonville, New York	6/21/2017	NY-2s1	IL-1
NY-2s2	ATGTTGCTGCTTCTCATACATCTCAGCATTTTGGTAATTTTCACTACCATCTACAAGATGCTCCCCGGCGGCATGTTCTCGAACACGGACCCATCCTGGGTCGATTGCCTGTACTTTTCGGCATCAACGCACACCACCGTGGGGTACGGGGACCTCACGCCCAAATCACCCGTTGCAAAACTCACGGCAACGGCACACATGCTGATCGTATTCGCGATCGTCATTTCTGGCTTCACGTTCCCGTGGTAA	Moodna Creek; Washingtonville, New York	6/21/2017	NY-2s1	IL-1
Pl_R_OLa (1)	ATGTTGCTGCTTCTCATACATCTCAGCATTTTGGTAATTTTCACTACCATCTACAAGATGCTCCCCGGCGGCATGTTCTCGAACACGGACCCATCCTGGGTCGATTGCCTGTACTTTTCGGCATCAACGCACACCACCGTGGGGTACGGGGACCTCACGCCAAAATCACCCGTGGCAAAACTCACGGCAACGGCACACATGCTGATCGTATTCGCGATCGTCATTTCTGGCTTCACGTTCCCATGGTAG	Platte River; Odessa, Nebraska	10/23/2017	Pl_R_Ola	IL-1
Somers_MT_m1 (1)	ATGTTGCTGCTTCTCATACATCTCAGCATTTTGGTAATTTTCACTACCATATACAAGATGCTCCCCGGCGGCATGTTCTCAAACACGGATCCGTCCTGGGTCGATTGCCTGTACTTTTCGGCATCAACGCACACCACCGTGGGGTACGGGGACCTCACGCCAAAATCACCCGTGGCAAAACTCACGGCAACGGCACACATGCTGATCGTATTCGCGATCGTCATTTCTGGCTTCACGTTCCCGTGGTAA	Flathead Lake; Somers, Montana	6/27/2017	Somers_MT_m1	IL-1
IL-M (1) [1]	ATGTTGCTGCTTATCATACATCTCAGCATTTTGGTAATTTTCACTACCATATACAAGATGCTCCCCGGCGGCATGTTCTCGAACACGGATCCGTCCTGGGTCGATTGCCTGTACTTTTCGGCATCAACGCACACCACCGTGGGGTACGGGGACCTCACGCCAAAATCACCCGTGGCAAAACTCACGGCAACGGCACACATGCTGATCGTATTTGCGATCGTCATTTCTGGCTTCACATTTCCGTGGTAG	Lake Zurich, Illinois	6/27/2017	IL-M	IL-M
Island-Lake_Medium (1) [1]	ATGTTGCTGCTCCTTATCCACGTGTGTATTTTGACAGTCTTCACGATTGTTTACAAGATGCTCCCTGGCGGCATGTTCTCTAACGCGGACCCGTCGTGGGTAGACTGCTTATACTTTGCCGCGTCGACTCACACCACAGTGGGGTACGGGGACCTCACCCCCAAATCGCCAGTGGCAAAGCTCACGGCGACGGCCCACATGTTGATCGTGTTCGCGATCATTATATCTAGCTTCACACTGCCATGGTAG	Island Lake, Western Nebraska	2018	Island-Lake_Medium	Island-Lake_Medium
Island-Lake_Small (1) [1]	ATGTTGCTGCTTATCATACATATCGTCATTCTTATAGTGTTCACTACCATCTACAAGATGCTCCCCGGCGGCATGTTCTCGAACACGGACCCGACTTGGGTTGATTGCCTGTACTTTTCGGCATCGACGCACACCACCGTGGGGTACGGAGATCTCACGCCCAAATCACCCGTGGCAAAGCTCACGGCAACGGCACACATGCTGATCGTATTCGCGATCGTCATTTCTGGCTTCACGTTTCCGTGGTAG	Island Lake, Western Nebraska	2018	Island-Lake_Small	Island-Lake_Small
mid_1.1 (1) [20]	ATGAAGCTGCTACTTTCACATATTGTTATTCTAATATGTTTCACCGTCGTGTACAAGATGCTCCCCGGTGGCATGTTCTCGGAAGCAGACCCGTCGTGGGTTGACTGTCTGTATTTCTCGACGGCAACACACACGACAACGGGCTACGGCGATCTAACGCCAGAAACCCCGGTGGCAAAACTCGTGACAACGGTGCACATGTTAACCGTGTTCATCATCGTTATTTCCGGCTTCACTGGCTTCGCATTATGGTAG	Germany	Oct-17	mid_1.1	mid_1.1
up_1.2m1 (19)	ATGAAGCTGCTACTTTCACATATCGTTATTCTAATATGTTTCACCGTCGTGTACAAGATGCTCCCCGGTGGCATGTTCTCGGAAGCAGACCCGTCGTGGGTTGACTGTCTGTATTTCTCGACGGCAACACACACGACAACGGGCTACGGCGATCTAACGCCAGAAACCCCGGTGGCAAAACTCGTGACAACGGTGCACATGTTAACCGTGTTCATCATCGTTATTTCCGGCTTCACTGGCTTCGCATTATGGTAG	Germany	Oct-17	up_1.2m1	mid_1.1
mid_1.2m	ATGAAGCTGCTACTTTCACATATCGTTATTCTAATATGTTTCACCGTCGTGTACAAGATGCTCCCCGGTGGCATGTTCTCGGAAGCAGACCCGTCGTGGGTTGACTGTCTGTATTTCTCGACGGCAACACACACGACAACGGGCTACGGCGATCTAACGCCAGAAACCCCGGTGGCAAAACTCGTGACAACGGTGCACATGTTAACCGTGTTCATCATCGTTATTTCCGGCTTCACTGGCTTCGCATTATGGTAG	Germany	Oct-17	up_1.2m1	mid_1.1
mid_1.2s2	ATGAAGCTGCTACTTTCACATATCGTTATTCTAATATGTTTCACCGTCGTGTACAAGATGCTCCCCGGTGGCATGTTCTCGGAAGCAGACCCGTCGTGGGTTGACTGTCTGTATTTCTCGACGGCAACACACACGACAACGGGCTACGGCGATCTAACGCCAGAAACCCCGGTGGCAAAACTCGTGACAACGGTGCACATGTTAACCGTGTTCATCATCGTTATTTCCGGCTTCACTGGCTTCGCATTATGGTAG	Germany	Oct-17	up_1.2m1	mid_1.1
mid_10.1L	ATGAAGCTGCTACTTTCACATATCGTTATTCTAATATGTTTCACCGTCGTGTACAAGATGCTCCCCGGTGGCATGTTCTCGGAAGCAGACCCGTCGTGGGTTGACTGTCTGTATTTCTCGACGGCAACACACACGACAACGGGCTACGGCGATCTAACGCCAGAAACCCCGGTGGCAAAACTCGTGACAACGGTGCACATGTTAACCGTGTTCATCATCGTTATTTCCGGCTTCACTGGCTTCGCATTATGGTAG	Germany	Oct-17	up_1.2m1	mid_1.1
mid_10.1s1	ATGAAGCTGCTACTTTCACATATCGTTATTCTAATATGTTTCACCGTCGTGTACAAGATGCTCCCCGGTGGCATGTTCTCGGAAGCAGACCCGTCGTGGGTTGACTGTCTGTATTTCTCGACGGCAACACACACGACAACGGGCTACGGCGATCTAACGCCAGAAACCCCGGTGGCAAAACTCGTGACAACGGTGCACATGTTAACCGTGTTCATCATCGTTATTTCCGGCTTCACTGGCTTCGCATTATGGTAG	Germany	Oct-17	up_1.2m1	mid_1.1
mid_10.1s2	ATGAAGCTGCTACTTTCACATATCGTTATTCTAATATGTTTCACCGTCGTGTACAAGATGCTCCCCGGTGGCATGTTCTCGGAAGCAGACCCGTCGTGGGTTGACTGTCTGTATTTCTCGACGGCAACACACACGACAACGGGCTACGGCGATCTAACGCCAGAAACCCCGGTGGCAAAACTCGTGACAACGGTGCACATGTTAACCGTGTTCATCATCGTTATTTCCGGCTTCACTGGCTTCGCATTATGGTAG	Germany	Oct-17	up_1.2m1	mid_1.1
mid_11.1m	ATGAAGCTGCTACTTTCACATATCGTTATTCTAATATGTTTCACCGTCGTGTACAAGATGCTCCCCGGTGGCATGTTCTCGGAAGCAGACCCGTCGTGGGTTGACTGTCTGTATTTCTCGACGGCAACACACACGACAACGGGCTACGGCGATCTAACGCCAGAAACCCCGGTGGCAAAACTCGTGACAACGGTGCACATGTTAACCGTGTTCATCATCGTTATTTCCGGCTTCACTGGCTTCGCATTATGGTAG	Germany	Oct-17	up_1.2m1	mid_1.1
mid_13.1L1	ATGAAGCTGCTACTTTCACATATCGTTATTCTAATATGTTTCACCGTCGTGTACAAGATGCTCCCCGGTGGCATGTTCTCGGAAGCAGACCCGTCGTGGGTTGACTGTCTGTATTTCTCGACGGCAACACACACGACAACGGGCTACGGCGATCTAACGCCAGAAACCCCGGTGGCAAAACTCGTGACAACGGTGCACATGTTAACCGTGTTCATCATCGTTATTTCCGGCTTCACTGGCTTCGCATTATGGTAG	Germany	Oct-17	up_1.2m1	mid_1.1
mid_13.1L2	ATGAAGCTGCTACTTTCACATATCGTTATTCTAATATGTTTCACCGTCGTGTACAAGATGCTCCCCGGTGGCATGTTCTCGGAAGCAGACCCGTCGTGGGTTGACTGTCTGTATTTCTCGACGGCAACACACACGACAACGGGCTACGGCGATCTAACGCCAGAAACCCCGGTGGCAAAACTCGTGACAACGGTGCACATGTTAACCGTGTTCATCATCGTTATTTCCGGCTTCACTGGCTTCGCATTATGGTAG	Germany	Oct-17	up_1.2m1	mid_1.1
mid_13.1s1	ATGAAGCTGCTACTTTCACATATCGTTATTCTAATATGTTTCACCGTCGTGTACAAGATGCTCCCCGGTGGCATGTTCTCGGAAGCAGACCCGTCGTGGGTTGACTGTCTGTATTTCTCGACGGCAACACACACGACAACGGGCTACGGCGATCTAACGCCAGAAACCCCGGTGGCAAAACTCGTGACAACGGTGCACATGTTAACCGTGTTCATCATCGTTATTTCCGGCTTCACTGGCTTCGCATTATGGTAG	Germany	Oct-17	up_1.2m1	mid_1.1
mid_5.1L1	ATGAAGCTGCTACTTTCACATATCGTTATTCTAATATGTTTCACCGTCGTGTACAAGATGCTCCCCGGTGGCATGTTCTCGGAAGCAGACCCGTCGTGGGTTGACTGTCTGTATTTCTCGACGGCAACACACACGACAACGGGCTACGGCGATCTAACGCCAGAAACCCCGGTGGCAAAACTCGTGACAACGGTGCACATGTTAACCGTGTTCATCATCGTTATTTCCGGCTTCACTGGCTTCGCATTATGGTAG	Germany	Oct-17	up_1.2m1	mid_1.1
mid_5.1L2	ATGAAGCTGCTACTTTCACATATCGTTATTCTAATATGTTTCACCGTCGTGTACAAGATGCTCCCCGGTGGCATGTTCTCGGAAGCAGACCCGTCGTGGGTTGACTGTCTGTATTTCTCGACGGCAACACACACGACAACGGGCTACGGCGATCTAACGCCAGAAACCCCGGTGGCAAAACTCGTGACAACGGTGCACATGTTAACCGTGTTCATCATCGTTATTTCCGGCTTCACTGGCTTCGCATTATGGTAG	Germany	Oct-17	up_1.2m1	mid_1.1
mid_5.1s1	ATGAAGCTGCTACTTTCACATATCGTTATTCTAATATGTTTCACCGTCGTGTACAAGATGCTCCCCGGTGGCATGTTCTCGGAAGCAGACCCGTCGTGGGTTGACTGTCTGTATTTCTCGACGGCAACACACACGACAACGGGCTACGGCGATCTAACGCCAGAAACCCCGGTGGCAAAACTCGTGACAACGGTGCACATGTTAACCGTGTTCATCATCGTTATTTCCGGCTTCACTGGCTTCGCATTATGGTAG	Germany	Oct-17	up_1.2m1	mid_1.1
mid_7.2	ATGAAGCTGCTACTTTCACATATCGTTATTCTAATATGTTTCACCGTCGTGTACAAGATGCTCCCCGGTGGCATGTTCTCGGAAGCAGACCCGTCGTGGGTTGACTGTCTGTATTTCTCGACGGCAACACACACGACAACGGGCTACGGCGATCTAACGCCAGAAACCCCGGTGGCAAAACTCGTGACAACGGTGCACATGTTAACCGTGTTCATCATCGTTATTTCCGGCTTCACTGGCTTCGCATTATGGTAG	Germany	Oct-17	up_1.2m1	mid_1.1
up_1.2m2	ATGAAGCTGCTACTTTCACATATCGTTATTCTAATATGTTTCACCGTCGTGTACAAGATGCTCCCCGGTGGCATGTTCTCGGAAGCAGACCCGTCGTGGGTTGACTGTCTGTATTTCTCGACGGCAACACACACGACAACGGGCTACGGCGATCTAACGCCAGAAACCCCGGTGGCAAAACTCGTGACAACGGTGCACATGTTAACCGTGTTCATCATCGTTATTTCCGGCTTCACTGGCTTCGCATTATGGTAG	Germany	Oct-17	up_1.2m1	mid_1.1
up_4.1L1	ATGAAGCTGCTACTTTCACATATCGTTATTCTAATATGTTTCACCGTCGTGTACAAGATGCTCCCCGGTGGCATGTTCTCGGAAGCAGACCCGTCGTGGGTTGACTGTCTGTATTTCTCGACGGCAACACACACGACAACGGGCTACGGCGATCTAACGCCAGAAACCCCGGTGGCAAAACTCGTGACAACGGTGCACATGTTAACCGTGTTCATCATCGTTATTTCCGGCTTCACTGGCTTCGCATTATGGTAG	Germany	Oct-17	up_1.2m1	mid_1.1
up_4.2	ATGAAGCTGCTACTTTCACATATCGTTATTCTAATATGTTTCACCGTCGTGTACAAGATGCTCCCCGGTGGCATGTTCTCGGAAGCAGACCCGTCGTGGGTTGACTGTCTGTATTTCTCGACGGCAACACACACGACAACGGGCTACGGCGATCTAACGCCAGAAACCCCGGTGGCAAAACTCGTGACAACGGTGCACATGTTAACCGTGTTCATCATCGTTATTTCCGGCTTCACTGGCTTCGCATTATGGTAG	Germany	Oct-17	up_1.2m1	mid_1.1
up_7.2	ATGAAGCTGCTACTTTCACATATCGTTATTCTAATATGTTTCACCGTCGTGTACAAGATGCTCCCCGGTGGCATGTTCTCGGAAGCAGACCCGTCGTGGGTTGACTGTCTGTATTTCTCGACGGCAACACACACGACAACGGGCTACGGCGATCTAACGCCAGAAACCCCGGTGGCAAAACTCGTGACAACGGTGCACATGTTAACCGTGTTCATCATCGTTATTTCCGGCTTCACTGGCTTCGCATTATGGTAG	Germany	Oct-17	up_1.2m1	mid_1.1
up_8.1	ATGAAGCTGCTACTTTCACATATCGTTATTCTAATATGTTTCACCGTCGTGTACAAGATGCTCCCCGGTGGCATGTTCTCGGAAGCAGACCCGTCGTGGGTTGACTGTCTGTATTTCTCGACGGCAACACACACGACAACGGGCTACGGCGATCTAACGCCAGAAACCCCGGTGGCAAAACTCGTGACAACGGTGCACATGTTAACCGTGTTCATCATCGTTATTTCCGGCTTCACTGGCTTCGCATTATGGTAG	Germany	Oct-17	up_1.2m1	mid_1.1
mid_3.1s (4) [4]	ATGAAGCTGCTACTTTCACATATCGTTATTCTAATATGTTTCACCGTTATTTACAAGATGCTCCCCGGTGGCATGTTCTCGGATGCGGACCCGTCGTGGTTTGACTGTCTGTATTTCTCGACGGCGACGCATACGACAACAGGCTACGGCGATCTAACGCCTAAGACGCCGGTGGCAAAACTCGTGACCACAGCGCATATGTTAACCGTTTTCGCGATCGTTATTTCCGGTTTCGCTGGCTTCAAGTTATGGTAG	Germany	Oct-17	mid_3.1s	mid_3.1s
mid_14.1	ATGAAGCTGCTACTTTCACATATCGTTATTCTAATATGTTTCACCGTTATTTACAAGATGCTCCCCGGTGGCATGTTCTCGGATGCGGACCCGTCGTGGTTTGACTGTCTGTATTTCTCGACGGCGACGCATACGACAACAGGCTACGGCGATCTAACGCCTAAGACGCCGGTGGCAAAACTCGTGACCACAGCGCATATGTTAACCGTTTTCGCGATCGTTATTTCCGGTTTCGCTGGCTTCAAGTTATGGTAG	Germany	Oct-17	mid_3.1s	mid_3.1s
mid_14.2	ATGAAGCTGCTACTTTCACATATCGTTATTCTAATATGTTTCACCGTTATTTACAAGATGCTCCCCGGTGGCATGTTCTCGGATGCGGACCCGTCGTGGTTTGACTGTCTGTATTTCTCGACGGCGACGCATACGACAACAGGCTACGGCGATCTAACGCCTAAGACGCCGGTGGCAAAACTCGTGACCACAGCGCATATGTTAACCGTTTTCGCGATCGTTATTTCCGGTTTCGCTGGCTTCAAGTTATGGTAG	Germany	Oct-17	mid_3.1s	mid_3.1s
mid_14.3	ATGAAGCTGCTACTTTCACATATCGTTATTCTAATATGTTTCACCGTTATTTACAAGATGCTCCCCGGTGGCATGTTCTCGGATGCGGACCCGTCGTGGTTTGACTGTCTGTATTTCTCGACGGCGACGCATACGACAACAGGCTACGGCGATCTAACGCCTAAGACGCCGGTGGCAAAACTCGTGACCACAGCGCATATGTTAACCGTTTTCGCGATCGTTATTTCCGGTTTCGCTGGCTTCAAGTTATGGTAG	Germany	Oct-17	mid_3.1s	mid_3.1s
mid_6.1 (1) [1]	ATGAAGCTGCTACTTTCACATATCGTTATTCTAATATGTTTCACCGTTATTTACAAGATGCTCCCCGGCGGCATGTTCTCGGATGCAGACCCGTCGTGGTTTGACTGTCTGTATTTCTCGACGGCGACGCATACGACAACAGGCTACGGCGATCTAACGCCCAAGTCGCCGGTGGCAAAACTCGTTACCACGGTGCATATGTTAACCGTGTTCGCGATCGTTATTTCCGGGTTCGCTGGCTTCAAGNTTCCATGGTAG	Germany	Oct-17	mid_6.1	mid_6.1
mid_6.2 (5) [5]	ATGAAGCTGCTACTTTCACATATCGTTATTCTAATATGTTTCACCGTTATTTACAAGATGCTCCCCGGCGGCATGTTCTCGGATGCAGACCCGTCGTGGTTTGACTGTCTGTATTTCTCGACGGCGACGCATACGACAACAGGCTACGGCGATCTAACGCCCAAGTCGCCGGTGGCAAAACTCGTTACCACGGTGCATATGTTAACCGTGTTCGCGATCGTTATTTCCGGGTTCGCTGGCTTCAAGTTTCCATGGTAG	Germany	Oct-17	mid_6.2	mid_6.2
mid_12.1	ATGAAGCTGCTACTTTCACATATCGTTATTCTAATATGTTTCACCGTTATTTACAAGATGCTCCCCGGCGGCATGTTCTCGGATGCAGACCCGTCGTGGTTTGACTGTCTGTATTTCTCGACGGCGACGCATACGACAACAGGCTACGGCGATCTAACGCCCAAGTCGCCGGTGGCAAAACTCGTTACCACGGTGCATATGTTAACCGTGTTCGCGATCGTTATTTCCGGGTTCGCTGGCTTCAAGTTTCCATGGTAG	Germany	Oct-17	mid_6.2	mid_6.2
mid_12.3L	ATGAAGCTGCTACTTTCACATATCGTTATTCTAATATGTTTCACCGTTATTTACAAGATGCTCCCCGGCGGCATGTTCTCGGATGCAGACCCGTCGTGGTTTGACTGTCTGTATTTCTCGACGGCGACGCATACGACAACAGGCTACGGCGATCTAACGCCCAAGTCGCCGGTGGCAAAACTCGTTACCACGGTGCATATGTTAACCGTGTTCGCGATCGTTATTTCCGGGTTCGCTGGCTTCAAGTTTCCATGGTAG	Germany	Oct-17	mid_6.2	mid_6.2
mid_12.3s	ATGAAGCTGCTACTTTCACATATCGTTATTCTAATATGTTTCACCGTTATTTACAAGATGCTCCCCGGCGGCATGTTCTCGGATGCAGACCCGTCGTGGTTTGACTGTCTGTATTTCTCGACGGCGACGCATACGACAACAGGCTACGGCGATCTAACGCCCAAGTCGCCGGTGGCAAAACTCGTTACCACGGTGCATATGTTAACCGTGTTCGCGATCGTTATTTCCGGGTTCGCTGGCTTCAAGTTTCCATGGTAG	Germany	Oct-17	mid_6.2	mid_6.2
mid_8.1	ATGAAGCTGCTACTTTCACATATCGTTATTCTAATATGTTTCACCGTTATTTACAAGATGCTCCCCGGCGGCATGTTCTCGGATGCAGACCCGTCGTGGTTTGACTGTCTGTATTTCTCGACGGCGACGCATACGACAACAGGCTACGGCGATCTAACGCCCAAGTCGCCGGTGGCAAAACTCGTTACCACGGTGCATATGTTAACCGTGTTCGCGATCGTTATTTCCGGGTTCGCTGGCTTCAAGTTTCCATGGTAG	Germany	Oct-17	mid_6.2	mid_6.2
mid_9.1 (1) [1]	ATGAAGCTGCTACTTTCACATATCGTTATTCTAATATGTTTCACCGTTATTTACAAGATGCTCCCCGGTGGCATGTTCTCGGATGCGGACCCGTCGTGGTTTGACTGTCTGTATTTCTCGACGGCGACGCATACGACAACAGGCTACGGCGATCTAACGCCTAAGTCGCCGGTGGCAAAACTCGTGACCACGGTGCATATGTTAACCGTTTTCGCGATCGTTATTTCCGGGTTCGCTGGCTTCAAGTTATGGTAG	Germany	Oct-17	mid_9.1	mid_9.1
MN08101 (1) [1]	ATGCTGCTTCTCCTGATACACATTGCCATATTGACATTCTTTACGGTCGTGTACAAGATGCTCCCCGACGGCGTGTTCTCGAACGGGGACCCGTCGTGGGTAGACTGCTTATACTTTTCCGCGTCCACTCACACCACCGTGGGATACGGGGACCTCACCCCCAAATCACCCGTGGCAAAACTCACGGCAACGGCCCATATGATGATCGTGTTCGCGATTGTAGTGTCTAGCTTCACGTTCCCGTGGTAG	Minnesota	2008	MN08101	MN08101
NEJV2 (2) [6]	ATGTTGCTGCTTATCATACATATCATCATTCTGATAGTGTTCACTACCATCTACAAGATGCTCCCCGGTGGTATGTTCTCGAACACGGACCCGACTTGGGTTGATTGCCTGTACTTTTCGGCATCGACGCACACCACTGTGGGGTACGGAGATCTCACGCCCAAATCACCCGTGGCAAAACTCACGGCAACGGCACACATGTTGATCGTATTCGCGATCGTCATTTCCGGCTTCACGTTTTCGTGGTAG	Rowe Bird Sanctuary; Gibbon, Nebraska	2008	NEJV2	NEJV2
Dismal_NE_s3	ATGTTGCTGCTTATCATACATATCATCATTCTGATAGTGTTCACTACCATCTACAAGATGCTCCCCGGTGGTATGTTCTCGAACACGGACCCGACTTGGGTTGATTGCCTGTACTTTTCGGCATCGACGCACACCACTGTGGGGTACGGAGATCTCACGCCCAAATCACCCGTGGCAAAACTCACGGCAACGGCACACATGTTGATCGTATTCGCGATCGTCATTTCCGGCTTCACGTTTTCGTGGTAG	Dismal River, Nebraska	5/26/2017	NEJV2	NEJV2
NEJV3 (1)	ATGTTGCTGCTTATCATACATATCATCATTCTGATAGTGTTCACTACCATCTACAAGATGCTCCCCGGCGGCATGTTCTCGAACACAGACCCGACTTGGGTTGATTGCCTGTACTTTTCGGCATCGACGCACACCACTGTGGGGTACGGAGATCTCACGCCCAAATCACCCGTGGCAAAACTCACGGCAACGGCACACATGTTGATCGTATTCGCGATCGTCATTTCCGGCTTCACGTTTTCGTGGTAG	Gudmundsen Ranch, NE	2008	NEJV3	NEJV2
MO3 (3)	ATGTTGCTGCTTATCATACATATCATCATTCTGATAGTGTTCACTACCATCTACAAGATGCTCCCCGGCGGCATGTTCTCGAACACAGACCCTACTTGGGTTGATTGCCTGTACTTTTCGGCATCGACGCACACCACCGTGGGGTACGGAGATCTCACGCCCAAATCACCCGTGGCAAAACTCACGGCAACGGCACACATGTTGATCGTATTCGCGATCGTCATTTCCGGCTTCACGTTTTCGTGGTAG	Lake Lotawana, Missouri	5/31/2017	MO3	NEJV2
NY2m	ATGTTGCTGCTTATCATACATATCATCATTCTGATAGTGTTCACTACCATCTACAAGATGCTCCCCGGCGGCATGTTCTCGAACACAGACCCTACTTGGGTTGATTGCCTGTACTTTTCGGCATCGACGCACACCACCGTGGGGTACGGAGATCTCACGCCCAAATCACCCGTGGCAAAACTCACGGCAACGGCACACATGTTGATCGTATTCGCGATCGTCATTTCCGGCTTCACGTTTTCGTGGTAG	Moodna Creek; Washingtonville, New York	6/21/2017	MO3	NEJV2
Verbena_VA_s3	ATGTTGCTGCTTATCATACATATCATCATTCTGATAGTGTTCACTACCATCTACAAGATGCTCCCCGGCGGCATGTTCTCGAACACAGACCCTACTTGGGTTGATTGCCTGTACTTTTCGGCATCGACGCACACCACCGTGGGGTACGGAGATCTCACGCCCAAATCACCCGTGGCAAAACTCACGGCAACGGCACACATGTTGATCGTATTCGCGATCGTCATTTCCGGCTTCACGTTTTCGTGGTAG	South fork of the Shenandoah River; Verbena, Virginia	7/1/2017	MO3	NEJV2
NTS1 (1) [1]	ATGTTGCTGCTTCTCATACATCTCAGCATTTTGGTAATTTTCACTGCCATCTACAAGATGCTGCCCGGCGGCATGTTCTCAAACACAGACCCGACTTGGGTCGATTGCCTGTACTTTTCGGCATCAACGCACACCACCGTGGGGTACGGGGACCTCACGCCAAAATCACCCGTGGCAAAACTCACGGCAACGGCACACATGCTGATCGTATTCGCGATCGTCATTTCTGGCTTCACGTTCCCGTGGTAG	Next to Smith Lake, Western Nebraska	2008	NTS1	NTS1
NY-2 (1) [4]	ATGTTGCTGCTTCTCATACATCTCAGCATTTTGGTAATTTTCACTACCATCTACAAGATGCTTCCCGGCGGCATGTTCTCGAACACGGACCCATCCTGGGTCGATTGCCTGTACTTTTCGGCATCAACACACACCACCGTGGGGTACGGGGACCTCACGCCAAAATCACCCGTGGCAAAACTCACGGCAACGGCACACATGCTGATCGTTTTCGCGATCGTCATTTCTGGCTTCACGTTCCCGTGGTAG	Moodna Creek; Washingtonville, New York	6/21/2017	NY-2	NY-2
Somers_MT_L3 (1)	ATGTTGCTGCTTCTCATACATCTCAGCATTTTGGTAATTTTCACTACCATCTACAAGATGCTCCCCGGCGGCATGTTCTCGAACACGGACCCATCCTGGGTCGATTGCCTGTACTTTTCGGCATCAACGCACACCACCGTGGGGTACGGGGACCTCACGCCAAAATCACCCGTGGCAAAACTCACGGCAACGGCACACATGCTGATCGTATTCGCGATCGTCATTTCTGGCTTCACGTTCCCGTGGTAA	Flathead Lake; Somers, Montana	6/27/2017	Somers_MT_L3	NY-2
Verbena_VA_L4 (2)	ATGTTGCTGCTTCTCATACATCTCAGCATTTTGGTAATTTTCACTACCATCTACAAGATGCTTCCCGGCGGCATGTTCTCGAACACGGACCCATCCTGGGTCGATTGCCTGTACTTTTCGGCATCAACGCACACCACCGTGGGGTACGGGGACCTCACGCCAAAATCACCCGTGGCAAAACTCACGGCAACGGCACACATGCTGATCGTTTTCGCGATCGTCATTTCTGGCTTCACGTTCCCGTGGTAG	South fork of the Shenandoah River; Verbena, Virginia	7/1/2017	Verbena_VA_L4	NY-2
Verbena_VA_m1	ATGTTGCTGCTTCTCATACATCTCAGCATTTTGGTAATTTTCACTACCATCTACAAGATGCTTCCCGGCGGCATGTTCTCGAACACGGACCCATCCTGGGTCGATTGCCTGTACTTTTCGGCATCAACGCACACCACCGTGGGGTACGGGGACCTCACGCCAAAATCACCCGTGGCAAAACTCACGGCAACGGCACACATGCTGATCGTTTTCGCGATCGTCATTTCTGGCTTCACGTTCCCGTGGTAG	South fork of the Shenandoah River; Verbena, Virginia	7/1/2017	Verbena_VA_L4	NY-2
Smith_L_La (3) [3]	ATGTTGCTGCTCCTTATCCACATGTGTATTTTGACATTCTTCACAGTTGTTTACAAGATGCTCCCTGGCGGCATGTTCTCTAACGCGGACCCGTCGTGGGTAGACTGCTTATACTTTGCCGCGTCGACTCACACCACGGTGGGGTACGGGGACCTCACCCCCAAATCGCCAGTGGCAAAGCTCACGGCGACGGCCCACATGTTGATCGTGTTCGCGATCATTATATCTAGCTTCACACTCCCATGGTAG	Smith Lake, Western Nebraska	10/22/2017	Smith_L_La	Smith_L_La
Smith-Lake_Medium-Plaque	ATGTTGCTGCTCCTTATCCACATGTGTATTTTGACATTCTTCACAGTTGTTTACAAGATGCTCCCTGGCGGCATGTTCTCTAACGCGGACCCGTCGTGGGTAGACTGCTTATACTTTGCCGCGTCGACTCACACCACGGTGGGGTACGGGGACCTCACCCCCAAATCGCCAGTGGCAAAGCTCACGGCGACGGCCCACATGTTGATCGTGTTCGCGATCATTATATCTAGCTTCACACTCCCATGGTAG	Smith Lake, Western Nebraska	2018	Smith_L_La	Smith_L_La
Island-Lake_Large-Plaque	ATGTTGCTGCTCCTTATCCACATGTGTATTTTGACATTCTTCACAGTTGTTTACAAGATGCTCCCTGGCGGCATGTTCTCTAACGCGGACCCGTCGTGGGTAGACTGCTTATACTTTGCCGCGTCGACTCACACCACGGTGGGGTACGGGGACCTCACCCCCAAATCGCCAGTGGCAAAGCTCACGGCGACGGCCCACATGTTGATCGTGTTCGCGATCATTATATCTAGCTTCACACTCCCATGGTAG	Island Lake, Western Nebraska	2018	Smith_L_La	Smith_L_La
SPRLa (1) [1]	ATGTTGCTGCTCCTTATACACATCGGTATTTTGGTATTTTTCACTATCGTGTACAAGATGCTCCCCGGCGGCATGTTCTCGAACACAGACCCTACTTGGGTCGATTGCCTGTACTTTTCGGCATCGACGCACACCACCGTGGGGTACGGAGATCTCACGCCCAAATCACCCGTGGCAAAACTCACGGCAACGGCACACATGTTGATCGTATTCGCGATCGTCATTTCCGGCTTCACGTTTCCGTGGTAG	South Platte River; Big Spring, Nebraska	10/23/2017	SPRLa	SPRLa
TN603 (1) [1]	ATGTTGCTGCTTCTCATACACCTCTGTATTTTGATAATTTTTACTACAATATACAAGATGTTGCCCGGAGGCATGTTCTCGAACACGGACCCGTCATGGATAGATTGCCTGTACTTCTCGGCATCAACGCACACCACCGTGGGGTACGGGGATCTCACGCCCAAATCGCCCGTGGCAAAACTCACAGCAACGGCACACATGCTGATCGTATTCGCGATCGTAATAACTGGCTTCACATTCCCGTGGTAA	Tennessee	2006	TN603	TN603
up_3.1 (4) [4]	ATGAAGCTGCTACTTTCACATATCGTTATTCTAATATGTTTCACCGTTATTTACAAGATGCTCCCCGGTGGCATGTTCTCGGATGCGGACCCGTCGTGGTTTGACTGTCTGTATTTCTCGACGGCGACGCATACGACAACAGGCTACGGCGATCTAACGCCTAAGTCGCCGGTGGCAAAACTCGTGACCACAGCGCATATGTTAACCGTTTTCGCGATCGTTATTTCCGGTTTCGCTGGCTTCAAGTTATGGTAG	Germany	Oct-17	up_3.1	up_3.1
up_5.2L	ATGAAGCTGCTACTTTCACATATCGTTATTCTAATATGTTTCACCGTTATTTACAAGATGCTCCCCGGTGGCATGTTCTCGGATGCGGACCCGTCGTGGTTTGACTGTCTGTATTTCTCGACGGCGACGCATACGACAACAGGCTACGGCGATCTAACGCCTAAGTCGCCGGTGGCAAAACTCGTGACCACAGCGCATATGTTAACCGTTTTCGCGATCGTTATTTCCGGTTTCGCTGGCTTCAAGTTATGGTAG	Germany	Oct-17	up_3.1	up_3.1
up_5.3m	ATGAAGCTGCTACTTTCACATATCGTTATTCTAATATGTTTCACCGTTATTTACAAGATGCTCCCCGGTGGCATGTTCTCGGATGCGGACCCGTCGTGGTTTGACTGTCTGTATTTCTCGACGGCGACGCATACGACAACAGGCTACGGCGATCTAACGCCTAAGTCGCCGGTGGCAAAACTCGTGACCACAGCGCATATGTTAACCGTTTTCGCGATCGTTATTTCCGGTTTCGCTGGCTTCAAGTTATGGTAG	Germany	Oct-17	up_3.1	up_3.1
up_5.3s2	ATGAAGCTGCTACTTTCACATATCGTTATTCTAATATGTTTCACCGTTATTTACAAGATGCTCCCCGGTGGCATGTTCTCGGATGCGGACCCGTCGTGGTTTGACTGTCTGTATTTCTCGACGGCGACGCATACGACAACAGGCTACGGCGATCTAACGCCTAAGTCGCCGGTGGCAAAACTCGTGACCACAGCGCATATGTTAACCGTTTTCGCGATCGTTATTTCCGGTTTCGCTGGCTTCAAGTTATGGTAG	Germany	Oct-17	up_3.1	up_3.1

^1^ Sequences submitted to the GenBank. The accession numbers for the *kcv* sequences in GenBank are MT560092–MT560194.
